# Radiation-induced lung injury: current evidence

**DOI:** 10.1186/s12890-020-01376-4

**Published:** 2021-01-06

**Authors:** Marisol Arroyo-Hernández, Federico Maldonado, Francisco Lozano-Ruiz, Wendy Muñoz-Montaño, Mónica Nuñez-Baez, Oscar Arrieta

**Affiliations:** 1 Head of Thoracic Oncology Unit, Unidad Funcional de Oncología Torácica, Instituto Nacional de Cancerología (INCan), Av. San Fernando #22, Sección XVI, Tlalpan, 14080 México City, CDMX México; 2 Departamento de Radioncología, Hospital Universitario HM Sanchinarro, Caracas, Venezuela

**Keywords:** Pneumonitis, Radiation pneumonitis, Radiation-induced lung injury, Lung cancer, Radiotherapy, Antineoplastic agents, Adverse effects

## Abstract

Chemo-radiotherapy and systemic therapies have proven satisfactory outcomes as standard treatments for various thoracic malignancies; however, adverse pulmonary effects, like pneumonitis, can be life-threatening. Pneumonitis is caused by direct cytotoxic effect, oxidative stress, and immune-mediated injury. Radiotherapy Induced Lung Injury (RILI) encompasses two phases: an early phase known as Radiation Pneumonitis (RP), characterized by acute lung tissue inflammation as a result of exposure to radiation; and a late phase called Radiation Fibrosis (RF), a clinical syndrome that results from chronic pulmonary tissue damage. Currently, diagnoses are made by exclusion using clinical assessment and radiological findings. Pulmonary function tests have constituted a significant step in evaluating lung function status during radiotherapy and useful predictive tools to avoid complications or limit toxicity. Systemic corticosteroids are widely used to treat pneumonitis complications, but its use must be standardized, and consider in the prophylaxis setting given the fatal outcome of this adverse event. This review aims to discuss the clinicopathological features of pneumonitis and provide practical clinical recommendations for prevention, diagnosis, and management.

## Background

For many years, radiotherapy (RT) has played an essential role in cancer treatments applied with curative intent or as an adjuvant treatment with chemotherapy or surgery [1]. It has also been used as a palliative remedy to alleviate symptoms in malignant and non-malignant disorders [2]. However, radiation-induced lung injury (RILI) is a severe complication of thoracic RT and embraces radiation pneumonitis (RP) and radiation fibrosis (RF) [3]. With the advent of new forms of radiotherapy techniques, such as Stereotactic Body Radiation Therapy (SBRT) and Intensity-modulated radiotherapy (IMRT), delivery of beam arrays to the tumor and surrounding tissues is more precise, reducing the incidence of lung toxicity. Risk factors related to the patient, treatment, and tumor can all influence the severity and extent of lung injuries. The challenge for diagnoses relies on the different clinical-radiological presentations and exclusion of alternative diagnoses, which explain the broad range of incidence rates (5%-58%) [4–7].

## Main text

### Predisposing factors

#### Treatment-related factors

1. *Radiation therapy*: RILI encompasses any lung damage due to lung exposure to ionizing radiation. This damage typically classified in early and late radiation toxicity could oscillate between asymptomatic and severe manifestations. Three common grading systems have used to stratify the severity of symptoms (see Table 1) [8]. In almost 40% of cases, conventional RT irradiates a considerable extension of healthy tissue surrounding the tumor. Therefore, new technologies of treatment delivery explored intensity-modulated RT (IMRT), volumetric arc radiotherapy (VMAT), and stereotactic body radiation therapy (SBRT) to highly individualized radiation treatment for primary tumors to minimize lung injury. In general, these techniques are well-tolerated, although major lung toxicity reported in up to 20% of cases [9, 10].
2. *Lung dose*: The total lung radiation dose is the main factor that predisposes to RP. The radiation doses delivered to the percentage of healthy lung tissue, receiving at least 20 Gy (V_20_), link to the development of lung toxicity [11, 12]. Additionally, low-dose parameters in the ipsilateral lung such as V_5_, V_10,_ and V_13_ are also related to the development of RP [13, 14]. Mean Lung Dose (MLD), or the average dose in proportion to total lung volume, has been reported as the best predictor of RP grade > 3 (OR, 1.002; 95% IC, 1.000–1.003; *p* = 0.03) [15].
3. *Fractionation*: For SBRT, the total dose of radiation treatment (40–70 Gy) is divided into large doses by fractions, thus shortening exposure time (1–2 weeks). When larger doses per fraction used (e.g., 50 Gy in 5 fractions), V_20_ > 10%, and a mean lung dose > 6 Gy are associated with a higher risk of RP grade ≥ 2 [16]. A recent multicenter trial compared the impact of the fractionation scheme—daily vs. every other day—on toxicities. There, the patients treated daily experienced significantly higher grade > 2 toxicities than the every-other-day patients (43 vs. 7%, *p* < 0.001) [17].
4. *Chemotherapy*: Chemotherapeutic agents like doxorubicin, taxanes, bleomycin, cyclophosphamide, vincristine, mitomycin, gemcitabine, and bevacizumab recognized as radiotherapy sensitizers due to their synergistic effect [18–20]. Taxane-induced pneumonitis seems to be higher when these agents combined with other cytotoxic drugs, especially gemcitabine.
5. *Concurrent treatment with chemo-radiotherapy (CCRT)*: Patients who receive combinations with cytotoxic agents have a higher frequency of lung toxicity compared with those who receive monotherapy (32 vs. 6%) [5, 21]. Remarkable, in an international cohort, radiation pneumonitis was increased in patients > 65 years treated with a platinum-taxanes combination [21]. However, a 2012 meta-analysis with over 1,600 patients demonstrated an increased OR of 1.6 (1.11–2.32) for RP in patients receiving concurrent treatment compared to sequential chemotherapy [22]. Furthermore, induction chemotherapy before concomitant treatment could increase the risk of RP, probably due to the prior radiosensitizing contribution of chemotherapy [23].
6. *Y*^*90*^* microsphere radioembolization*: Radioembolization delivers directed intraarterial doses of yttrium-90 (Y^90^) for treating hepatocellular carcinoma and liver metastases. RP is one of the most critical risks of radioembolization. Significant shunting to the lungs may result in lung toxicity, which has related to lung shunt fraction of more than 13% [24, 25]. During the planning of Y^90^ radioembolization, the assessment of splanchnic and pulmonary shunting may predict lung deposition of microspheres, and the risk for RP [26].
7. *Immunotherapy*: Pneumonitis due to immunotherapy is a rare but potentially life-threatening immune-related Adverse Event (irAE) derived from a direct cytotoxic effect, oxidative stress, and immune-mediated injury. The time elapsed between immunotherapy and the presence of pneumonitis varies from 1 to 24 months [27, 28]. Combined treatment with anti-PD1/PD-L1 increases pneumonitis risk by 1.5–2 times, moreover, anti-PD-1/PD-L1 combined with anti-CTLA-4 risk is 10% compared to the use of monotherapy (3%) [29, 30]. Furthermore, combination strategies of nivolumab and tyrosine-kinase inhibitors concomitantly or sequentially increase the risk of pneumonitis considerably [31]. Pneumonitis recurrence after rechallenge occurs in 60% of patients, and some cases even more extensively and severely [32–34]. Safety in re-treatment with immune checkpoint inhibitors does not depend entirely on the severity of the initial adverse event.

**Table 1:** Grading system for radiation induced lung toxicity

	RTOG	CTCAE v. 5.0	SWOG
Grade 0	No changes	No changes	Normal
Grade 1	Asymptomatic or mild symptoms	Asymptomatic, only radiological, or tomographic findings	Radiographic changes, symptoms do not require steroids
Grade 2	Moderate symptoms of pneumonitis (severe cough) and radiographic changes (radiographic patches)	Symptomatic, does not interfere with daily activities	Steroids required or tap of effusion
Grade 3	Severe symptoms of pneumonitis, dense radiographic changes	Symptomatic, interferes with daily activities, requires supplemental O2	Oxygen required
Grade 4	Symptoms of severe respiratory failure requiring assisted ventilation or continuous O2	Threatens life, requires mechanical ventilation support	Requires assisted ventilation
Grade 5	Death-related late effects of radiotherapy	Death related severe pneumonitis	–

*RTOG* Radiation Therapy Oncology Group [63], *CTCAE v. 5.0* Common Terminology Criteria for Adverse Events version 5.0 [64], *SWOG* Southwest Oncology Group criteria [65]

#### Tumor-related factors

1. *Tumor location*: Disease location in the mid-lower lung is more strongly associated with the development of RP [22, 35, 36]. Two possible explanations are 1) tumor motion may require a large lung radiated volume [37] and 2) patients can have inhomogeneous perfusion before RT due to previous lung diseases, tissue infiltrates, and cancer involvement. Thus, radiation may cause damage, which decreases perfusion and increases tissue density.
2. *Tumor histology and staging*: No association has yet been reported between tumor histology and RP development, while the effect of tumor stage in predicting RP risk is controversial.
3. *Tumor volume*: Patients with a higher tumor volume usually have a more significant lung and surrounding radiated volume. The most crucial factor that influences the development of RP is thus related to the percentage of lung radiated volume [13, 38].

#### Patient-related factors

1. *Smoking*: Smoking is deleterious for survival, noteworthy the effect on the increased risk of developing RILI is not clear. There is evidence that smoking could play a protective role in patients who underwent radiotherapy [39]. In the other hand, 20% higher risk of developing pneumonitis has reported in smokers and current smokers [40]; contrary to the protective effect observed by Vogelius et al. (OR 0.7, *p* < 0.06) [22], and the higher incidence of RP in nonsmokers compared to former smokers (37 vs. 14%) [35, 39]. The protective effect of tobacco reinforced by the fact that smoking-damaged lungs have less radiosensitivity to injury due to non-functional airspaces and non-vital tissue presence. However, this information should not encourage tobacco consumption in cancer patients.
2. *Comorbid conditions*: The most common pre-existing conditions in lung cancer patients are chronic obstructive pulmonary disease (COPD) and interstitial lung disease (ILD). COPD is found in 40–70% of patients and is an independent mortality prognostic factor in lung cancer patients; furthermore, an increase of RP has described. [41, 42] Patients with ILD often develop lung cancer within five years (15.4%) [41]. ILD has related to a considerably increased risk of any grade and fatal RP in patients with pre-radiotherapy computerized tomography (CT) scan findings [43]. Moreover, no differences in any grade of RP or mortality associated with ILD in subclinical patients who underwent SBRT [44]. COPD and ILD patients with RILI might be more symptomatic due to their impaired lung function, which leaves them highly susceptible to small changes [38, 45]. It also seems that in a lower grade, pulmonary emphysema has related to the development of RP in non-small cell lung cancer (NSCLC) patients [46, 47].
3. *Age and sex*: Women have a lower lung volume than men, yet in most studies, the association with RP risk has not been demonstrated [35]. Older patients may be more likely than younger ones to have a lower general functional status, comorbidities, and reduced lung function, which may explain the high RP risk [46]. Although the cut-off point for defining young versus old patients could bias the estimated effect, elderly patients > 70 reported to have worse RP (6% vs. 1%), often associated with RP grade ≥ 2 [39]. Therefore, RP risk should be considered in patients of all ages, but other factors must also be considered.
4. *Genetic phenotypes*: Genetic variation in crucial genes in DNA replication and repair, inflammation, and oxidative stress pathways, may either ameliorate or exacerbate the effects of a given radiation dose on the lungs. The risk of RP increases as the number of unfavorable genotypes increases (see Table 2).

**Table 2:** Genes associated with lung radiation toxicity

Study		Gene associated with lung toxicity year	Gene function	References
Pu et al.	2014	RPCDC2 (rs10711) and (rs1871445) DDX58 (rs11795343) and (rs7865082) FGF5 (rs3733336)	Inflammation	[87]
		ETS2 (rs2298560) LIMS1 (rs12469016)		
		GHR (rs4292454) TFEB (rs13202921)		
Wen et al.	2014	RP LIN28B (rs314280)	RNA binding protein	[88]
		LIN28B (rs314276)		
Pang et al.	2013	HSPB1 (rs2868371)	Oxidative stress pathways	[89]
Xiong et al.	2013	ATM (rs189037) and (rs228590)	DNA repair	[90]
Kelsey et al.	2013	XRCC1 (rs25487) DNA repair BRCA1 (rs16942)	DNA repair	[91]
Mak et al.	2012	MTHFR (rs1801131)	Oxidative stress pathways	[92]
Xu et al.	2015	LIG4 (rs1805388)	DNA repair	[93]
Yin et al.	2012	VEGF (rs2010963) and (rs3025039)	Angiogenesis	[94]
Niu et al.	2012	TGF-β1 (rs11466345)	Inflammation	[95]
Wang et al.	2008	P53 Arg72Pro	Cell-cycle regulation, apoptosis, and DNA repair	[96]
Hildebrandt et al.	2010	RPIL1A (rs1800587/rs17561) IL-8 (rs4073)	Inflammation	[97]
		TNF (rs1799724) TNFRSF1B (rs1061622)		
		MIF (rs7555622) IL4 (rs2243250) IL4R		
		(rs2070874) IL13 (rs10800925) IL13 (rs20541)		
		NFKBIA (rs1799983) NOS3 (rs1799983)		
Zhang et al.	2010	ATM (rs189037) and (rs373759)	DNA repair	[98]
Yuan et al.	2009	TGF-β1 (rs1982073)	Inflammation	[99]

Transforming growth factor-beta 1 (TGF-*β*1), Deoxyribonucleic-acid (DNA), Ribonucleic-acid (RNA)

### Pathophysiology of radiation-induced pneumonitis

**Fig. 1:**
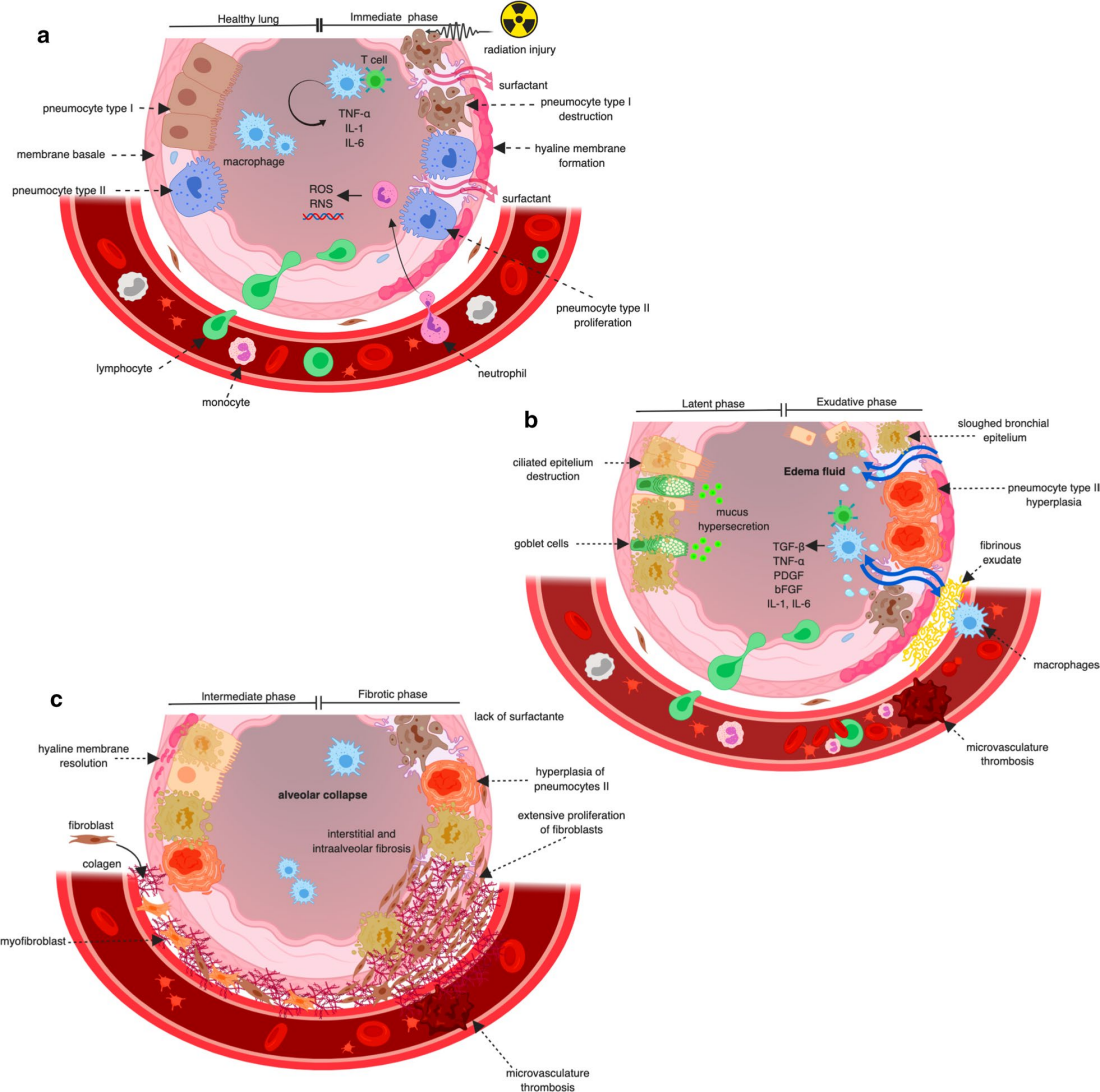
Pathophysiology and signaling pathways involved in pulmonary irradiation. **a** Healthy lung and radiation lung injury: early phase; **b** Radiation lung injury: latent and exudative phases; **c** Radiation lung injury: intermediate and fibrotic phases. TNF-α: tumor necrosis factor-α; IL-1: interleukin-1; IL-6: interleukin-6; PDGF-*β*: platelet-derived growth factor-*β*;bFGF: basic fibroblastic growth factor; ROS: release of reactive oxygen species; RNS: reactive nitrogen species; TGF-*β*1: transforming growth factor-beta. Created in Biorender. https://biorender.com/

Radiation induces a loss of the alveolar barrier function by destroying epithelial and endothelial cells. The inflammatory response induces a cycle of increased inflammation, vascular permeability, and cytokine release within days or weeks [32, 48]. Macrophage accumulation and activation contribute to the development of hypoxia, stimulating the production of reactive oxygen species and reactive nitrogen species (ROS/RNS) and proinflammatory, profibrogenic and proangiogenic cytokines that perpetuate a non-healing tissue response that leads to chronic radiation injury [49]. The changes induced by radiation can be divided into five phases according to exposure time (see Fig. 1).

1. *Early phase* begins within hours or days of RT and consists of vascular congestion that induces leukocyte infiltration, pneumocytes type I apoptosis, and intra-alveolar edema. The first cytokines released within two weeks post-radiotherapy and include the following: tumor necrosis factor-α (TNF-α), interleukin-1 (IL-1), interleukin-6 (IL-6), high-molecular-weight mucin-like antigen KL-6, platelet-derived growth factor-β (PDGF-β), and basic fibroblastic growth factor (bFGF) [50]. A second wave activates 6–8 weeks post-radiation and is associated with increased oxidative damage to the DNA, hypoxia, decreased lung perfusion, and increased expression of transforming growth factor-beta 1 (TGF-β1) [51].
2. *Latent phase* characterized by augmented secretions due to the proliferation of respiratory goblet cells and ciliary cell malfunction. It accompanied by tracheal-bronchial hypersecretion and degenerative changes in the alveolar epithelium and endothelium [52].
3. *Exudative phase* is also known as the clinical RP phase and occurs 3–12 weeks after RT exposure. It consists of epithelial and endothelial detachment that causes alveolar collapse accompanied by a narrowing of the pulmonary capillaries and microvascular thrombosis. The desquamation of pneumocytes and the alveolar secretion of a fibrin-rich exudate contribute to the formation of hyaline membranes. Also, the alveolar restitution characterized by the proliferation of pneumocyte type II re-epithelializing the alveolar basement membrane [53].
4. *Intermediate phase* refers to the dissolution of hyaline membranes, which occurs following the synthesis of collagen by fibroblasts that migrate and proliferate in the alveolar walls. The importance of TGF-β1 expression is based on the influx of fibroblasts and their conversion to myofibroblasts, which produces lung fibrosis. This condition, in turn, leads to hypoxia, which induces the release of both profibrogenic and proangiogenic factors, so this cycle continues until it reaches the ultimate stage of chronic lung disease [52].
5. *Fibrotic phase* can appear after six months of radiation exposure. It evolved biologically for several years and characterized by hyperplastic pneumocytes, increased myofibroblasts, and extensive collagen depositions in the pulmonary interstitium and alveoli. These deposits lead to the collapse of alveolar spaces, thus reducing pulmonary volume [54].

### Radiation recall pneumonitis

Radiation recall pneumonitis (RRP) is an acute inflammatory response in a previously irradiated lung after the administration of systemic antineoplastic agents [55]. It often begins after exposure to the triggering agent but may occur at any time during treatment. The time elapsed from treatment onset to the presence of RRP ranges from hours to years, and the severity does not correlate with the interval of time between radiotherapy and antineoplastic treatment [56]. Taxanes and anthracyclines are the most common drugs related to RRP. Like nivolumab or durvalumab, immune-checkpoint inhibitors have reported higher incidences of severe pneumonitis in the irradiated lungs of previously treated NSCLC patients [34, 57]. Chemotherapy agents may be more likely than immunotherapy to be related to RRP; thus, the rate and severity of this phenomenon will change as the use of immunotherapy and targeted therapies become the preferable upfront treatment in the majority of clinical scenarios.

### Clinical approach

Clinical diagnosis often complicated due to the presence of non-specific symptoms that may be related to either pre-existing pathologies or malignant diseases. Thoracic radiation produces acute and late clinical manifestations after lung injury. RILI can divide into an acute phase (< 6 months), also called RP, and a chronic phase (> 6 months) known as RF [58–60]. Some authors refer to the acute phase as the development of pneumonitis that lasts from 4 to 12 weeks after radiation [61]. In acute lung injury, patients may experience an exacerbation of previous respiratory symptoms or new clinical manifestations, such as dyspnea and coughing, which occur in 20–40% of cases [62]. Severe signs and symptoms are less frequent but involve hypoxemia and respiratory failure. Conversely, the chronic phase manifests itself as progressive respiratory insufficiency or progressed severity of previous symptoms, particularly dyspnea. Non-specific signs and symptoms include tachypnea, cyanosis, crackles or pleural rub under thorax examination, chest pain, malaise, and occasionally, fever. Moreover, less frequent RP clinical features may include hemoptysis, airway obstruction, pulmonary vascular damage, bronchitis, respiratory infections, pleural effusion, and pneumothorax. The physical examination finding may include pleural friction rub, moist rales, and signs of consolidation.

### Grading

There are different grading systems defined pneumonitis severity, regarding clinical, radiological changes, and type of treatment or medical support required. The most commonly used are the Radiation Therapy Oncology Group (RTOG) [63], the Common Terminology Criteria for Adverse Events v. 5.0 (CTCAE v 5.0) [64], and the Southwest Oncology Group Criteria (SWOG) [65]. RP events scored by the RTOG and CTCAE v 5.0 standards classify symptoms and image findings into five grades, while the SWOG score focuses on treatment requirements (see Table 1).

### Image abnormalities

There is a broad spectrum of imaging findings after radiation injury that can mimic other etiologies. The extension of RP damage varies according to the radiological image observed. It can display from scarce patchy lesions within the irradiated field with fading of the pulmonary vasculature to very extensive lesions with well-defined areas of consolidation. A chest X-ray is the first tool used in approaches to RP. Radiographic lung damage sees in lung volumes irradiated at doses under 20 Gy. Newer techniques like VMAT or IMRT have the potential to deliver lower doses of radiation distributed in lung volumes. V5, V10, and V15 dose constraints are correlated with RP, however, V5 has linked with symptomatic RP [66]. VMAT and IMRT should be employed as the prefer radiation techniques in addition to strict dosimetric constraints. CT-scan is more sensitive than chest radiography for detecting subtle lung injury following radiation treatment. Different CT findings have described in RP: diffuse ground-glass opacity, modified conventional, mass-like consolidation with volume loss and traction bronchiectasis, and scar-like [48, 67, 68].

Conventional RF generally involves the entirety of irradiated lung tissue. Acute image findings are usually confined to the radiation field and appear as ground-glass opacities that may progress to the organizing or consolidation phases [61, 68]. Also, it can be present as ipsilateral pleural effusion and atelectasis. Lung opacities may gradually resolve over six months without radiological sequelae or with minimal damage [51]. Late radiological findings result from unresolved acute RP. They include diminished volume, linear scarring, consolidation, and traction bronchiectasis.

**Fig. 2:**
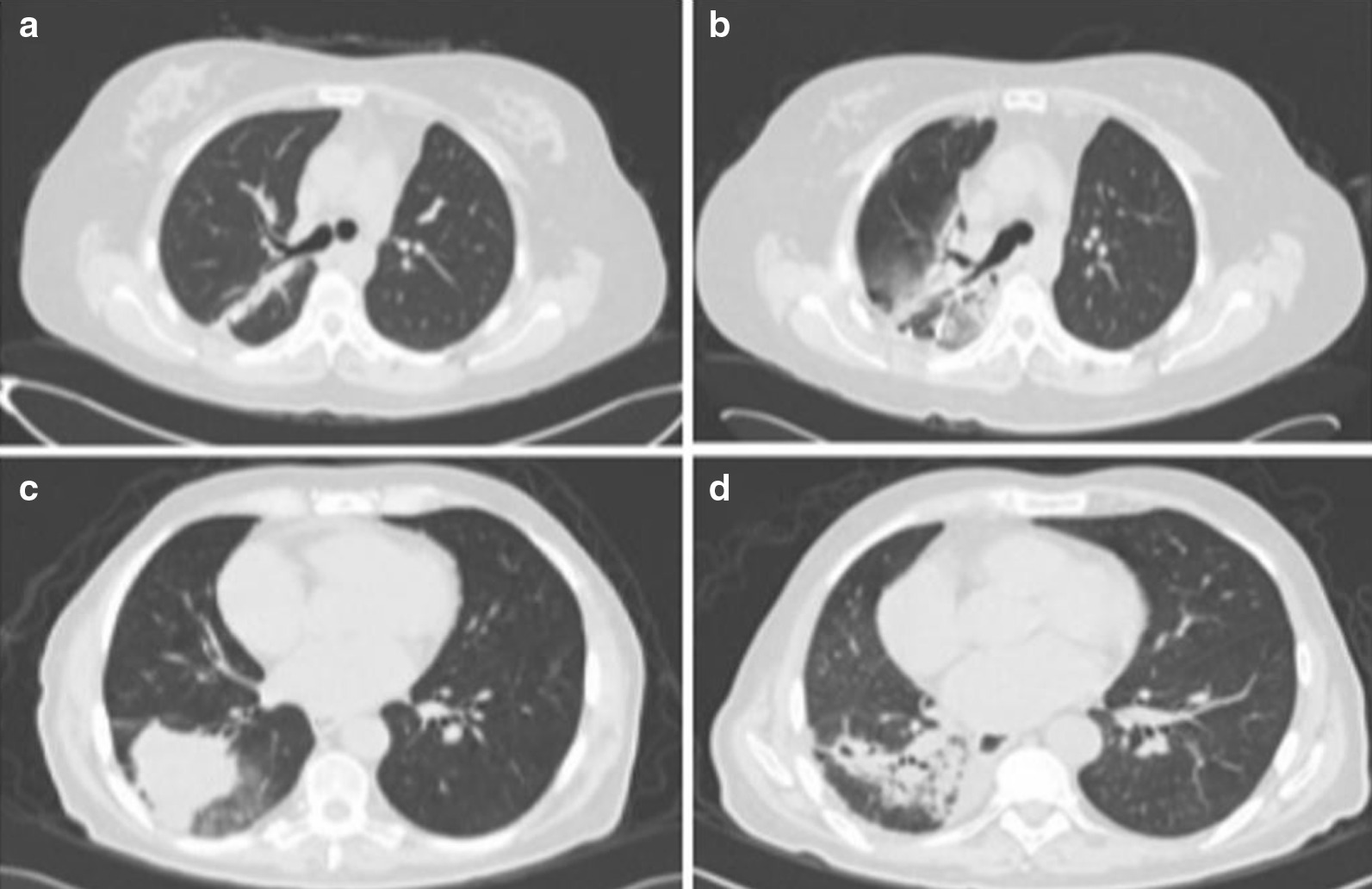
Imaging findings after radiation injury that can mimic other etiologies. **a** Axial images of a CT scan performed 1-week before RT and three months post-RT; **b** A ground-glass opacity image with atelectasis; **c** Axial images of a CT scan with tumor image in the lower lobe before RT and six months post-RT; **d** Consolidation, bronchiectasis, and a scar-like pattern.

In some cases, pleural thickening and mediastinum traction may be observed [48]. In advanced stages, shrinkage of the pulmonary parenchyma and areas of fibrosis along the path of the radiation beam may be visible. RF and loss of lung volume may continue to evolve for as long as 24 months, resulting in architectural distortion [53] (see Fig. 2). Kouloulias et al. proposed a CT grading scale to categorize lung damage's extension and characteristics in 5 scores, but further studies are required to validate its use [50].

### Pulmonary function tests

Reduced baseline mechanical function measured by spirometry, plethysmography, and gas exchange abnormalities on the diffusion lung capacity of carbon monoxide test (DLCO) has elucidated as predictive factors for RP [16, 54]. The lung is not a uniform organ, and patients with lung cancer experience distinct changes in response to RT in different functional regions [37]. After RT, a mild decrease in the forced expiratory volume in 1 s (FEV_1_) may indicate minor development of bronchial obstruction due to tissue swelling. Declines in forced vital capacity (FVC) and total lung capacity (TLC) indicate a degree of lung stiffening since both are indicators of lung compliance. As larger volumes are irradiated, a more significant decrease in lung compliance seems to appear, affecting FVC, TLC, and, subsequently, FEV_1_. A reduction of DLCO after RT refers to an interstitial lung tissue damage that compromises the gas transfer through the alveolocapillary membrane. The six-minute walking test (6-MWT) is a useful predictor tool for RP in patients undergoing thoracic irradiation for lung carcinoma. A 6-MWT/FVC ratio below 4 ft/l predicts chronic RP [69]. A prospective study in NSCLC patients evaluating lung function with a broad range of lung function tests showed that a reduction in baseline FEV_1_ (*p* = 0.02) and DLCO (*p* = 0.02), together with an increase in the fractional exhaled nitric oxide (FeNO) test (*p* = 0.04), correlate with the development of RP [70]. Likewise, a reduction in lung function observed before symptoms appear in patients with radiation-induced lung injury. Pulmonary function tests have proven to be useful tools for the early detection of lung tissue changes secondary to radiation [71, 72].

### Diagnosis and treatment

Diagnoses of RP require clinical correlation and exclusion of the most common pathologies that mimic lung toxicity, mainly disease progression and infection. It is essential to differentiate these conditions to provide adequate treatment and proper management. RP is a diagnosis established by clinical suspicion or radiological findings; in selected cases, a lung biopsy helped to rule out confounders.

**Fig. 3:**
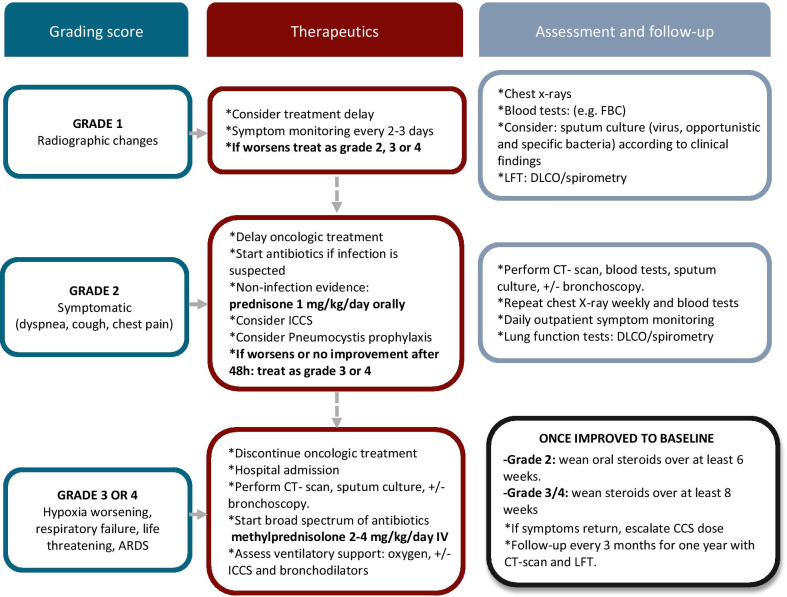
RP Management and follow-up. *ARDS* Acute respiratory distress syndrome, *IV* intravenous, *MMF* Mycophenolate mofetil, *FBC* full blood count, *DLCO* Diffuse capacity of carbon monoxide, *CCS* corticosteroids, *ICCS* inhaled-corticosteroids, *CT-scan* computed tomography scan, *LFT* lung function tests

The mainstay of treatment in acute pneumonitis consists of administering a systemic corticosteroid cycle at high doses for symptomatic patients or those with sub-acute onset, grade ≥ 2; however, few controlled studies have evaluated the efficacy of RP treatment in humans. Oral prednisone generally prescribed at 1–2 mg/kg/day before tapering down over 3–12 weeks, depending on institutional recommendations, is an option [73]. For grades 3–4, intravenous corticosteroids equivalent to methylprednisolone at 2–4 mg/kg/day tapered over six weeks is recommended. Glucocorticoids reduce inflammation and inhibit TNF-induced nitric oxide-mediated endothelial cell and lymphocyte toxicity. Prophylaxis for Pneumocystis Jirovecci recommended for patients with risk factors and those taking high dose corticosteroids (see Fig. 3) [74, 75]. The use of inhaled corticosteroids ensures that the highest dose is deposited in the airway, thus decreasing side effects. Although there is no fixed treatment dosage, inhaled steroids have shown efficacy as a treatment option for RP grade 2 [76]. However, few studies have assessed the efficacy of inhaled steroids in cancer patients. It is well-known that patients with chronic disease (radiation fibrosis) are unlikely to benefit from glucocorticoid therapy [60].

### Prophylaxis and new treatment options

Several strategies have tested prophylactically or in the early stages of RP to avoid fibrosis development; however, despite positive results, none of the following are standard of care in the current clinical practice. Pentoxifylline has immunomodulatory and anti-inflammatory properties mediated by the suppression of TNF-α and IL-1, which may play a role in treating of RF. The effects of pentoxifylline at a dose of 400 mg, taken orally three times a day for eight weeks, have proven to improve clinical signs, symptoms, and a reduction in lung fibrosis [77]. It significantly reduces the presence of fibrosis with alpha tocopherol (vitamin E) for six months [78–80].

Amifostine is a radioprotector agent that functions via free radical scavenging. In animal models, it diminishes the concentration of TGF-β1 [81]. Two meta-analyses evaluated and verified the benefit of amifostine at reducing the risk of RP vs. placebo/non-treatment, without affecting tumor response [82, 83].

Angiotensin-converting enzyme inhibitors (ACE-inhibitors) exhibit significant antifibrotic activity against collagen accumulation in the lungs; however, its effectiveness has proved in retrospective trials [84, 85]. Since excess collagen synthesis is a crucial histopathologic feature of RF, other drugs, such as colchicine, penicillamine, statins, and interferon-gamma, may also have the potential to modify the progression of fibrosis.

Recently, nintedanib has emerged as a promising form of treatment and prophylaxis for RF (NTC 02452463, NTC02496585), since it showed benefits reducing the annual FVC decline in patients with idiopathic pulmonary fibrosis that shares similar pathophysiology [86].

## Conclusions

Pneumonitis is a potentially life-threatening adverse event caused by treatment that has become more common since the introduction of new, personalized therapies. Some progress has made in elucidating its multifactorial mechanism, and various risk factors have identified to implement preventive strategies. However, there are still enormous gaps in our knowledge, particularly about the role of specific cytokines as predictive factors, the use of accurate diagnostic tests, and the advent of new prophylactic and curative therapeutic solutions. Achieving a better understanding of the pathophysiology of pneumonitis is still the focus of efforts to develop new treatments and prophylactic drugs. These facts highlight the essential role of patient follow-up when receiving RT. The principal focus of future research should be developing individualized clinical and lung function monitoring, specific therapies, preventing, and reducing sequelae.

## Data Availability

Not applicable.

## Abbreviations

RILI: Radiotherapy induced lung injury
RP: Radiation pneumonitis
RF: Radiation fibrosis
RT: Radiotherapy
SBRT: Stereotactic body radiation therapy
IMRT: Intensity-modulated radiotherapy
VMAT: Volumetric arc radiotherapy
MLD: Mean lung dose
CCRT: Chemo-radiotherapy
Y^90^: Yttrium-90
irAE: Immune-related adverse event
COPD: Chronic obstructive pulmonary disease
ILD: Interstitial lung disease
CT: Computerized tomography
NSCLC: Non-small cell lung cancer
ROS: Reactive oxygen species
RNS: Reactive nitrogen species
TNF-α: Tumor necrosis factor-α
IL-1: Interleukin-1
IL-6: Interleukin-6
PDGF-β: Platelet-derived growth factor-β
bFGF: Basic fibroblastic growth factor
TGF-β1: Transforming growth factor-beta 1
RRP: Radiation recall pneumonitis
RTOG: Radiation Therapy Oncology Group
CTCAE v 5.0: Common terminology criteria for adverse events v.5.0
SWOG: Southwest oncology group criteria
DLCO: Diffusion lung capacity of carbon monoxide test
FEV_1_: Forced expiratory volume in 1s
FVC: Forced vital capacity
TLC: Total lung capacity
6-MWT: Six-minute walking test
FeNO: Fractional exhaled nitric oxide
ACE-inhibitors: Angiotensin-converting enzyme inhibitors
ARDS: Acute respiratory distress syndrome
IV: Intravenous
MMF: Mycophenolate mofetil
FBC: Full blood count
DLCO: Diffuse capacity of carbon monoxide
CCS: Corticosteroids
ICCS: Inhaled-corticosteroids
LFT: Lung function tests
